# Cross-cultural adaptation and validation of the German Central Sensitization Inventory (CSI-GE)

**DOI:** 10.1186/s12891-021-04481-5

**Published:** 2021-08-18

**Authors:** Michel Klute, Marjan Laekeman, Katrin Kuss, Frank Petzke, Angela Dieterich, Andreas Leha, Randy Neblett, Steffen Ehrhardt, Joachim Ulma, Axel Schäfer

**Affiliations:** 1 Pain Clinic, Department of Anaesthesiology, University Medical Center, Georg August University of Göttingen, Robert-Koch-Str. 40, 37075 Göttingen, Germany; 2 Physiological Psychology, Otto-Friedrich- University of Bamberg, Bamberg, Germany; 3 Department of General Practice/Family Medicine, Philipps University Marburg, Marburg, Germany; 4 Physiotherapy, Faculty of Health, Safety, Society, Furtwangen University, Furtwangen, Germany; 5 Department of Medical Statistics, University Medical Center Göttingen, Göttingen, Germany; 6 PRIDE Research Foundation, Dallas, TX USA; 7 Faculty of Social Sciences, City University of Applied Sciences, Bremen, Germany; 8 Clinic for Pain Medicine Bremen, Rotes-Kreuz-Krankenhaus Bremen, Bremen, Germany; 9 Faculty of Social Work and Health, University of Applied Science and Art, Hildesheim, Germany

**Keywords:** Central sensitization inventory, Central sensitization, Central sensitivity syndromes, CSI, CSI-GE, Chronic pain, Cross-cultural adaptation, Psychometric validation

## Abstract

**Background:**

The Central Sensitization Inventory (CSI) is a screening tool designed to detect symptoms related to Central Sensitization (CS) and Central Sensitivity Syndromes (CSS) by measuring the degree of related phenomena. The objective of this study was to create a German, culturally-adapted version of the CSI and to test its psychometric properties.

**Methods:**

A German version of the CSI (CSI-GE) was developed, culturally-adapted, and pretested for comprehensibility. The psychometric properties of the resulting version were validated in a clinical study with chronic pain and pain-free control subjects. To assess retest reliability, the CSI-GE was administered twice to a subgroup of patients. Structural validity was tested using factor analyses. To investigate construct validity a hypotheses testing approach was used, including (1) correlations between the CSI-GE and several other well-established questionnaires as well as (2) an investigation of the CSI-GE discriminative power between different subgroups of participants believed to have different degrees of CS.

**Results:**

The CSI-GE showed excellent reliability, including high test-retest characteristics. Factor analyses confirmed a bi-factor dimensionality as has been determined previously. Analysing construct validity 6 out of 11 hypotheses (55%) were met. CSI-GE scores differentiated between subgroups according to expectations. Correlations between CSI-GE scores and other questionnaires suggested that none of the correlated constructs was identical, but there was overlap with other questionnaires based on symptom load. Several correlations did not fit with our current understanding of CS.

**Conclusion:**

The CSI-GE appears to be a reliable tool for measuring CS/CSS-related symptomatology. Whether this implies that the CSI-GE measures the degree of CS within an individual subject remains unknown. The resulting score should be interpreted cautiously until further clarification of the construct.

**Supplementary Information:**

The online version contains supplementary material available at 10.1186/s12891-021-04481-5.

## Introduction

Chronic Pain is often related to a multitude of underlying factors, which can trigger, contribute to, and maintain it [1, 2]. Recently, a new classification for chronic pain was added to the ICD-11 (International Classification of Diseases). It introduced a whole chapter on chronic pain conditions that are now understood as primary health problems in themselves [3, 4]. Central Sensitization (CS) appears to be an important feature for the development and maintenance of many of these chronic pain conditions, irrespective of other etiological aspects [5–7]. The International Association for the Study of Pain [8] defines CS as “*increased responsiveness of nociceptive neurons in the central nervous system to their normal or subthreshold afferent input*”. No gold standard for the diagnosis of CS exists, so it is difficult to assess its presence and magnitude [5, 9, 10]. There have been many different attempts to objectively quantify CS [11], including Quantitative Sensory Testing (QST) [12] and imaging techniques [11]. However, these tools are complex, time-consuming and expensive [12, 13].

Yunus [14] postulated that CS is a common feature in a number of insufficiently understood syndromes, often called MUS (medically unexplained symptoms) due to the lack of structural pathology. He suggested that these disorders be renamed Central Sensitivity Syndromes (CSS) and introduced the idea that CS may be a common feature causing similar and overlapping symptoms in these syndromes. In addition to a lack of structural pathology, most CSSs objectively share a lowered pain threshold and heightened pain sensitivity [15] which is a main feature of CS [16].

Mayer et al. [17] developed a patient-reported screening instrument called the Central Sensitization Inventory (CSI) to help identify and quantify CS/CSS-related symptomology. The concept of the CSI is based on Yunus’s [14] model of CSSs, in which different conditions with different phenotypes share overlapping symptoms related to CS. These symptoms were extracted from the CSS conglomerate via literature search and condensed in one questionnaire. The instrument has attracted growing attention and has been translated, culturally adapted and validated in different languages [18]. Some inconsistent results have been found regarding the dimensionality of the CSI in these initial validation studies (Supplement 1). To help settle the question of CSI dimensionality, a multi-country study with over 2000 subjects determined a bi-factor model, with one general factor of “CS-related symptoms” and four latent factors [19]. The validation of the underlying construct in different countries with different translations has been done in different ways or not addressed at all.

In this project, the CSI was first translated into the German language, pretested for comprehensibility, and culturally adapted. Then its psychometric properties were tested, including internal consistency, dimensionality, construct validity, and discriminative ability in a group of subjects with a broad spectrum of chronic musculoskeletal pain disorders and a separate group of pain-free control subjects.

## Methods

### Translation, cultural adaption and pre-test

This study consisted of two parts. First, the original version of the CSI was forward (English to German) and backward (German translation back into English) translated, and cross-culturally adapted into German by an expert translation committee following the multistep approach recommended by the American Association of Orthopaedic Surgeons Outcomes Committee [20, 21]. A pretest was performed with 15 patients with chronic pain recruited at the pain clinic of the University Medical Center Göttingen. Based on the pretest results, the expert committee adapted two items, resulting in the final German version of the CSI (CSI-GE). The translation and cross-cultural adaptation of the CSI-GE has previously been presented elsewhere [22]. A detailed description of the steps involved in the translation is presented in Supplement 2. The German version of the CSI (CSI-GE) is presented in Supplement 3.

### Clinical study for the psychometric validation

Secondly, a multicentre clinical study was conducted to assess the psychometric properties of the CSI-GE with four recruiting partners: the multidisciplinary pain clinic at the University Medical Center Göttingen; the pain clinic at Rotes Kreuz Krankenhaus Bremen; the MVZ Endokrinologikum (an outpatient rheumatology practice) based in Göttingen; and an outpatient practice for pain medicine, also located in Göttingen. The multicentre study design followed the COSMIN recommendations [23, 24] and was chosen to ensure the needed number of patients with a broad spectrum of pain-related diagnoses.

### Ethical approval

For each involved institution ethical approval through the responsible ethics committee was provided: Ethics Committee of the University Medical Center Göttingen (15/09/2017) (including the pretests), Ethics Committee of the Ärztekammer Bremen (Antrags-Nr. 666–11/04/2019), and the Ethics Committee of the Ärztekammer Niedersachsen (Grae/055/2019).

The validation study was registered at the German Clinical Trials Register (DRKS-ID: DRKS00015252). All participants signed informed consent.

### Participants

Patients with diverse musculoskeletal pain disorders were included in this study. Inclusion criteria were chronic pain for at least 3 months, sufficient physical and cognitive ability to participate, sufficient knowledge of the German language, and age older than 18 years. Exclusion criteria were psychiatric disease with pain as the main symptom (somatoform disorders, severe depression), initial or unstable phase of a rheumatological disease, or a primarily neuropathic pain component. All patients with chronic pain seeking routine care during the recruitment period were considered for participation and screened for eligibility by the responsible physicians of the individual case and at least one member of the study team. Eligible patients were informed about the study and asked for consent to participate.

In addition, a healthy control group (HC) was recruited from the general population via local contacts. For that purpose, members of a local choir were asked to participate in the study. To broaden the age range for the control group, students from the University Göttingen and acquaintances of the study team were asked to participate as well. The primary inclusion criteria were that they reported neither acute nor chronic pain. This was determined by an additional questionnaire asking for the presence of other than every day kind of pain (initial question of the brief pain inventory [25]) or pain-related conditions and was also explicitly assessed by a member of the study team during the recruitment. All other inclusion and exclusion criteria for the control participants were the same as for the clinical group.

To determine the appropriate sample size, we followed the recommendations for a factor analysis (FA), suggesting 4 to 10 participants per item [24] and more than 100 participants overall [26]. Interpreting this rule of thumb conservatively, 250 participants were needed because the CSI includes 25 items. We aimed to recruit 250 chronic pain patients (CPP) and 50 healthy control (HC) subjects.

### Subgrouping of the chronic pain patients (CPP)

The chronic pain group was classified into five subgroups as described below. The subgrouping was based on clinical reasoning and on the main pain-related diagnosis and distribution of pain as used for classification in KEDOQ-Schmerz, a German pain register project for chronic pain based on the data provided by the widely-used German pain questionnaire [27, 28]. The German pain questionnaire includes multiple well-validated instruments. It serves for initial clinical assessment as well as progress assessment of pain patients. The results were available for all patients prior to inclusion into the present study. Diagnoses were made based on the results of the German pain questionnaire as well as an in-person interdisciplinary assessment. Complicated cases with unclear classifications were discussed and resolved with the responsible physicians assigned to the individual cases and two of the authors (FP, MK), who were mainly responsible for the subgrouping. The following subgroups were presumed to represent different levels of CS [7] on a continuum, with Fibromyalgia representing the highest levels and regional chronic pain the lowest levels:

- The fibromyalgia (FMS) subgroup included patients with fibromyalgia as the primary diagnosis. Fibromyalgia was determined by the preliminary 2011 ACR criteria, including a full clinical assessment [29].
- Multisite chronic pain (MCP) included patients with a diverse spectrum of chronic pain disorders, with pain in at least three body regions.
- Regional chronic pain (RCP) included patients with a localized pain disorder in one well-defined body region (e.g., pain in the hand or foot only).
- Chronic back and/or neck pain (CBNP) included patients with a chronic pain syndrome in the back and/or neck region.
- In addition, patients with Rheumatoid arthritis in remission (RAR) were included if their rheumatologist clinically determined they were in disease remission at the time of their consultation. The level of CS in this subgroup was presumed to be lower than in the multisite chronic pain and fibromyalgia subgroups. All patients within this (RAR) subgroup were recruited at the outpatient rheumatological practice and evaluated by both the rheumatologist and one member of the study team (MK).

### Questionnaires

#### Central sensitization inventory (CSI)

The CSI consists of two parts (A and B). Part A includes 25 items which assess typical symptoms associated with CS/CSS. Patients rate the degree of these symptoms on a five-point Likert scale ranging from never to always (never = 0, rarely = 1, sometimes = 2, often = 3, always = 4). The summation of all single item scores results in a total score ranging from 0 to 100. Part B inquires about 10 previously-diagnosed disorders in the patient’s medical history, including seven common CSSs and three other conditions linked to CS/CSS. Part B of the CSI only aims to provide additional information and is not scored [17, 18]. We decided to quantify a sum score for part B by adding one point for each positively answered question resulting in a score ranging from 0 to 10. This score was used to correlate CSI parts A and B.

#### Pain sensitivity questionnaire minor (PSQm)

The PSQm is a condensed version of the PSQ which assesses individual subjective sensitivity to pain. The instrument includes 7 questions rated between 0 and 10. The mean rating of the 7 questions is used as the resulting score. The PSQm has previously demonstrated high correlations with sensitivity to experimental pain in healthy controls [30] and chronic pain patients [31].

#### Depression anxiety stress scale (DASS)

The DASS includes three scales which measure symptoms of depression, anxiety, and stress. The instrument contains 21 items (7 on each scale). All items can be rated from 0 to 3, higher scores represent more severe symptoms. The German version was validated in 2015 and has demonstrated acceptable psychometric properties for screening for depression, anxiety, and stress in patients with chronic pain [32].

#### Patient health questionnaire 15 (PHQ15)

The PHQ15 is a self-administered instrument that contains 15 items which assess the severity of somatic symptoms, indicating the individual degree of somatization. It has been translated into the German language and validated [33]. We used 13 items of the PHQ15 in order to have the same total score for women and men and to skip one problematic item that has often not been answered. Therefore, the questions asking for “menstrual cramps or other problems with your periods” and “pain or problems during sexual intercourse” were excluded. This resulted in a maximal score of 26 points for the adapted version.

#### painDETECT questionnaire

The painDETECT is a screening tool to detect a neuropathic pain component in chronic pain patients [34]. It has been developed and validated in collaboration with the German Research Network on Neuropathic Pain. As previously described, we used only one subscale of the painDETECT that includes 7 questions assessing neuropathic pain symptoms [35]. Questions are rated between never (0) and very strongly (5) resulting in a total score ranging from 0 to 35.

#### Pain Catastrophizing scale (PCS)

The PCS focuses on the quantification of catastrophizing attitudes and thoughts towards pain [36]. It contains 13 items which are rated on a 5-point Likert scale, resulting in an overall score ranging from 0 to 52. It has previously been translated and validated in the German language [37].

#### Fibromyalgia survey questionnaire (FSQ)

The FSQ is a self-administered tool to identify FMS in survey research without a physical examination. It has been validated for the German population [38] and consists of two subscales. One is the Widespread Pain Index (WPI), which assesses pain or tenderness at 19 different body parts, resulting in a total score between 0 and 19. The other subscale is the Somatic Severity Score (SSS). It captures the somatic symptom burden by inquiring about fatigue, trouble thinking, tiredness after waking up, pain in the lower abdomen, depression, and headache. SSS total scores range from 0 to 12.

#### Marburger questionnaire on habitual well-being (MFHW)

The MFHW is a short Questionnaire capturing perceived general wellbeing by addressing positive thoughts in 7 questions. The total score ranges from 0 to 35. Higher scores indicate a higher degree of habitual well-being [39].

#### Veterans Rand 12 (VR12)

The VR 12 [40] measures health-related quality of life and is very similar to the SF-12 (12-Item Short Form Health Survey) [41]. It results in two scores measuring the physical (physical composite summary - PCS) and the mental (mental composite summary - MCS) status separately. Higher scores indicate higher quality of physical or mental health-related quality of life. The VR12 has previously been translated and validated in the German language [42].

#### Graded chronic pain scale (VonKorff scale)

Von Korff et al. [43] introduced an instrument to grade the severity of chronic pain on a scale of 0 to 4 based on pain intensity and pain-related disability. The instrument also includes a numeric rating scale (0–10), which asks for the mean pain intensity within the last 4 weeks. The score of that subscale was used to indicate each subject’s perceived pain intensity.

#### Mainz pain staging system (MPSS)

The MPSS by Gerbershagen distinguishes three increasing degrees of pain chronicity by its associated features (like medication use, distribution and variability of pain, prior treatments) [1]. Throughout the past decades, it has been validated several times for different patient groups [44, 45]**.**

In addition to the questionnaires, we included a question about the duration (in months) of each patient’s chronic pain status.

### Data collection

Study participants had the choice to complete the questionnaires during a hospital admission, outpatient visit, or at home (postage-prepaid envelope provided). All returned questionnaires were checked for missing items and participants were contacted to provide the missing answers. Participants were instructed in a standardized manner to complete missing items including the option not to answer.

### Test-retest reliability

To analyse test-retest reliability, a time-interval of 2 weeks between two measurement occasions and a sample size of *n* = 50 was determined adequate, assuming an ICC (intraclass-correlation-coefficient) of 0.8 with a 95% CI of ±0.1 [24]. A subgroup of pain patients (*n* = 56) received a second questionnaire for completion of the CSI-GE 2 weeks after the initial study visit. They were provided with a prepaid envelope and reminded after 3 weeks if the envelope had not been received by then. The test-retest time interval of 2 weeks, which has previously been recommended by de Vet et al. [24], seemed long enough to avoid patients remembering previous answers and short enough to avoid changes in health status affecting answers.

## Statistical analysis

To assess the psychometric properties of the CSI-GE part A, both reliability and validity were investigated. Reliability was tested by analysing internal consistency and test-retest reliability. For assessment of the structural validity, both exploratory and confirmatory factor analyses (FA) were used to analyse the dimensionality of the questionnaire. To assess the construct validity, the relationship with other well-established clinical variables and questionnaires was analysed. In addition, to assess the discriminative validity, the ability of the CSI-GE to differentiate among different patient subgroups, believed to have different levels of CS (as described above) was investigated. The normality and data distributions were assessed by the skewness, kurtosis, histograms, Q-Q Plots, and Kolmogorov-Smirnov as well as Shapiro-Wilk tests. Demographic variables are described by means and standard deviation. Overall sex differences within the different subgroups were analysed using Fisher’s-Exact-Test. Age differences were analysed using a Kruskal-Wallis-Test followed post hoc analysis for every specific subgroup comparison.

Floor and ceiling effects were determined by examining the prevalence of participants scoring the lowest and highest possible score on the CSI-GE sum score. As proposed by McHorney and Tarlov [46] the effects were considered relevant and problematic if observed in more than 15% of the participants.

### Reliability

Internal consistency was evaluated using Cronbach’s α. To evaluate test-retest reliability, the intraclass-correlation-coefficient_2,1_ (ICC_2,1_ – two-way random, absolute agreement, single measures) [47] was calculated for the CSI-GE part A overall sum score. Following KOO and Li [48] ICC values < 0.5 were considered poor, 0.5 to 0.75 moderate, 0.75 to 0.9 good, and > 0.9 excellent. The limits of agreement were analysed using a Bland Altman Plot. Therefore, the differences were plotted against the averages of every patient measurement pair included in the test-retest analysis. Standard Error of Measurement (SEM) and Smallest Detectable Change (SDC) were computed, using formulas proposed by de Vet et al. [24]. SEM was calculated by dividing the standard deviation of the difference between timepoint one and timepoint two, by the square root of two (SEM = SD_difference_/ $\sqrt{2}$). The SDC was calculated following the formula SDC= ± 1.96*SEM* $\sqrt{2}$.

### Validity

#### Structural validity

Structural validity was analysed using FA. Exploratory factor analysis (EFA) using Maximum-Likelihood-Extraction with Promax-Rotation was carried out with data from the chronic pain sample to assess whether there would be a different factor structure than described in previous validation studies. Confirmatory factor analyses (CFA) were performed to assess the fit of three previously published models to our dataset, including both subject groups (CPP and HC). We assessed the fit to the original model by Mayer et al. [17] with four latent factors: “physical symptoms “(items 1, 2, 5, 6, 8, 9, 12, 14, 17, 18, 22), “emotional distress “(items 3, 13, 15, 16, 23, 24), “headache” (items 4, 7, 10, 19, 20) and “urological symptoms” (items 11, 21, 25); the fit to the 1-factor model by Cuesta-Vargas et al. [49] with one “general factor” that included all 25 items; and the fit to the bifactor model by Cuesta-Vargas et al. [19] that included both one general factor “CS related symptoms” (all 25 items) and 4 latent factors based on the four factors described by Mayer et al. [17]. The factor structure of all models can be found in Table 3. Diagonally weighted least squares estimation was used with listwise deletion of cases with missing information. Latent factors were standardized, allowing free estimation of all factor loadings. The Tucker-Lewis Index (TLI) and the root mean square error of approximation (RMSEA) with its 90% confidence interval were reported for each confirmatory model fit. Following Browne and Cudeck [50] a model fit was judged excellent for RMSEA< 0.05, good for RMSEA< 0.08, mediocre for RMSEA< 0.1, and poor for RMSEA> 0.1. Following Schermelleh-Engel et al. [51], a model fit was judged acceptable for TLI > 0.95 and good for TLI ≥ 0.97. Additionally, the factor loadings and (if applicable) factor correlations were analysed, each with the *p*-values from the respective significance tests. An ANOVA-type χ^2^ difference test between the nested models was performed.

#### Construct validity – hypotheses testing

To assess construct validity 11 different hypotheses (Table 6) were formulated, and potential outcomes were postulated. This hypotheses testing used two approaches: (1) predefined differences between relevant subgroups based on differences in CSI-GE total score and (2) predefined correlations of the CSI-GE score with questionnaires that were selected based on potential clinical characteristics of CS as an overall construct. As proposed by Prinsen et al. [52] construct validity was considered satisfactory if ≥75% of the hypotheses were met as predefined. In addition to the 11 hypotheses, further group comparisons and correlations with other instruments were analysed but not included in the hypotheses testing approach as a clear prediction of the outcome and relationship to the CSI-GE was not possible prior to our analysis. Therefore, the purpose of these additional measures was explorative aiming to further characterize the CSI-GE construct.

The discriminative power of the CSI-GE was assessed by examining differences among the six subgroups (FMS, MCP, CBNP, RAR, RCP, HC) using a Kruskal-Wallis test and Dunn-Bonferroni post-hoc-tests for nonparametric data. Bonferroni-Correction for multiple comparisons was used. A conservative non-parametric statistical approach was chosen for this analysis due to the violation of normal distribution and heterogeneity of variance between the different subgroups.

Pairwise correlations between total scores on each questionnaire and on the CSI-GE were calculated using Kendall’s τ as a non-parametric correlation coefficient. Only data from the CPP were used, and pairwise deletion of missing cases was applied. Tests against the null hypothesis of no correlation were performed. Correction for multiple testing was done using Holm’s procedure. Following Cohen [53], correlations were considered small for Pearson’s correlation coefficient *r* > 0.1, medium for *r* > 0.3, and large for *r* > 0.5. To use these categories for the differently scaled Kendall’s τ, we translated these to the scale of Kendall’s τ, using the formula given by Kendall [54]. Consequently, correlations with τ > 0.16 were considered small, correlations with τ > 0.48 were considered medium and correlations with τ > 0.82 were considered large.

The significance level was set to alpha = 5% for all statistical tests. The pairwise correlations with other questionnaires and the CFA were performed with the statistic software R version 3.6.1 [55] using the R-package lavaan version 0.6.5 [56] for the confirmatory factor analysis. Analyses for the demographics, the reliability, group comparisons, and the EFA were performed using IBM SPSS Statistics for Macintosh, Version 26.0, Released 2019 (Armonk, NY, USA: IBM Corp.).

## Results

### Demographic data and distribution

At the end of the recruitment phase, 346 individuals had been recruited to the study. Thirty-six datasets could not be included for different reasons listed in Table 1, resulting in 310 valid datasets for analysis. All exclusions were discussed and confirmed prior to analyses in a data validation meeting by the authors. The 310 analysed datasets consisted of 247 CPP and 63 HC. The retest was given to 56 CPP. However, only 45 valid retests were available for analyses. Reasons for the loss of 11 datasets are also listed in Table 1*.* The allocation of the 310 datasets to subgroups and the corresponding demographics are shown in Table 2. The allocation shows only a small group with regional pain; most patients had low back or neck pain, or some form of multisite pain.

**Table 1:** Exclusions of datasets

	CPP	HC	Total CPP + HC	Retest
Agreed to participate, but did not send back the questionnaire	19	5		6
Excluded because:				
Diagnosis was not certain or in contrast to the recruitment criteria	7			
Missing items in CSI part A	4			1
Relevant Pain condition		1		
Significant health status changes within the retest time interval				4
Total of excluded datasets	30	6	**36**	**11**

*Abbreviations*: *CPP* chronic pain patients, *HC* healthy controls

**Table 2:** Subgroups & demographic figures

Different grouping	n	Age			Sex	CSI-GE part A			CSI-GE part B		
		mean	SD	Min/Max	%female	mean	SD	Min/Max	mean	SD	Min/Max
All individuals	310	54.7	13.1	22/81	70.0	38.5	17.3	4/77	1.6	1.75	0/9
HC	63	49.4	15.6	22/78	58.7	18.4	9.3	4/41	0.3	0.8	0/3
CPP											
All CPP	247	56.1	12.0	22/81	72.9	43.6	15.0	7/77	1.9	1.8	0/9
FMS	37	55.0	12.3	27/77	94.6	54.9	11.7	22/77	3.6	2.0	0/9
MCP	63	56.8	11.4	37/81	74.6	49.8	11.8	11/74	2.5	1.6	0/8
CBNP	83	54.8	12.5	25/79	66.3	40.9	13.5	13/75	1.6	1.5	0/6
RAR	47	59.8	10.4	31/77	66.0	35.9	14.4	9/63	0.66	1.0	0/4
RCP	17	51.7	13.7	22/74	70.6	31.4	15.5	7/58	1.0	1.1	0/4

*Abbreviations*: *HC* healthy controls, *CPP* chronic pain patients, *FMS* fibromyalgia syndrome, *MCP* multisite chronic pain, *CBNP* chronic back and neck pain, *RAR* rheumatoid arthritis in status of remission, *RCP* regional chronic pain, *n* number of cases, *SD* standard derivation, *Min/Max* minimum/maximum

As shown in Table 2, the mean age was 54.7 years for all individuals, with the HC group showing the lowest (49.4) and the RAR group showing the highest (59.8) mean age. The respective Kruskal-Wallis-Test showed the presence of an overall significance age difference (*p* = 0.01). However, post hoc comparisons showed that this age difference was only significant between the HC and RAR group. Seventy percent of the individuals were female, with 50.8% in the HC group and 94.6% in the FMS Group. Fisher’s exact test (*p* = 0.002) indicated that overall, sex was not distributed similarly within all subgroups. Correlating the CSI-GE sum score with age (τ = 0.19; *p* = 0,62) and sex (τ = 0.22; *p* < 0,001) showed only a weak correlation with age (*p* = n.s.) and weak correlation with sex. The mean CSI-GE total score in the total CPP sample was 43.6 and in the HC sample was 18.4. The FMS group had the highest mean CSI-GE score (54.9) and the HC group had the lowest. The FMS group also reported the highest number of summed responses (3.6) on CSI-GE part B, and the HC group reported the lowest number of responses (0.3).

No floor (sum score = 0) or ceiling effects (sum score = 100) occurred with total CSI-GE scores. The lowest score was 4 points by two participants (0,65%), and the highest score was 77 points by one participant (0,32%).

### Reliability

**Fig. 1:**
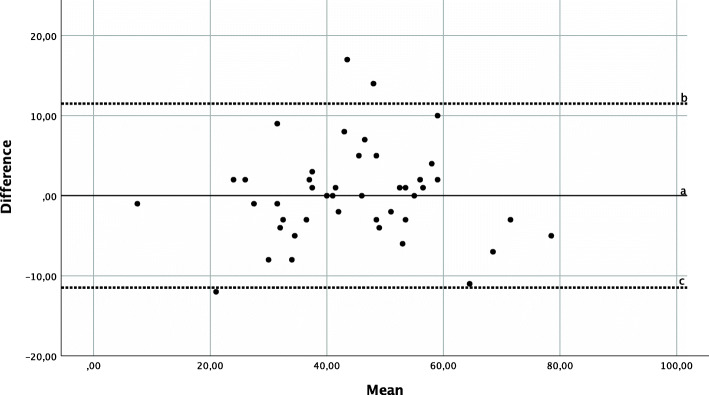
Limits of agreement – Bland Altman Plot: The difference (y-axis) = CSI-GE sum score time point 1 – CSI-GE sum score time point 2 was plotted against the mean (x-axis) = (CSI-GE sum score time point 1 + CSI-GE sum score time point 2)/2 for every patient. Line a (=0.022) represents the mean systematic difference between the two time points. Line b represents a + 1.96*standard deviation _difference_ (=11.508). Line c represents a-1.96*standard deviation _difference_ (= − 11.464). Therefore, lines b and c show the limits of agreement enclosing 95% of the patients in between

Reliability analysis of the CSI-GE yielded a Cronbach’s α of 0.928, which can be considered high [24]. Excellent test-retest reliability was demonstrated by an ICC_2,1_ of 0.917 (95% KI: 0.855; 0.954) for the overall sum score of the CSI-GE, with a mean test-retest time interval of 18.42 (min. 14/max. 32) days. The limits of agreement can be observed in Fig. 1. The SEM amounted to 4.144 and the SDC to ± 11.486.

### Validity

#### Exploratory factor analysis

**Fig. 2:**
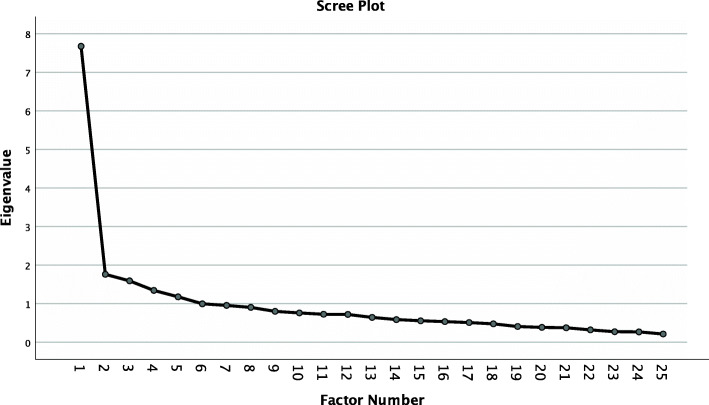
Scree plot EFA

The Kaiser-Meyer-Olkin criterion (0.88) and Bartlett’s test (*p* < 0.001) showed an overall good [57] suitability of the data for the EFA. The EFA found five possible factors with an Eigenvalue > 1. The Eigenvalue decreased strongly from the first factor (7.67) to the second factor (1.76) (scree plot Fig. 2), which indicated a 1-factor model. After rerunning the analysis and extraction of one factor, the 1-factor model was able to explain 27.94% of the variance. As demonstrated in Table 3, four items (4 = 0.39; 10 = 0.35; 11 = 0.38; 24 = 0.27) did not load above 0.4 on that single factor.

**Table 3:** Factor analysis - summary of different factor models

CSI-GE item	Factor analysis										
	EFA (*n* = 247)	CFA (*n* = 310)									
	1-factor model	4 factor model TLI 0.99 RMSEA 0.06 (90%-CI: [0.05; 0.07]) *x*^2^(269) = 553.09, *p* < 0.001				1-factor model TLI 0.98 RMSEA 0.08 (90%-CI: [0.07; 0.08]) χ^2^(275) = 756.39, *p* < 0.001	bifactor model TLI 0.99 RMSEA 0.05 (90%-CI: [0.04; 0.06]) χ^2^(250) = 430.6, *p* < 0.001				
	General factor	Physical symptoms	Emotional distress	Headache	Urological symptoms	General factor	General factor	Physical symptoms	Emotional distress	Headache	Urological symptoms
	Cron. α 0.928	Cron. α 0.893	Cron. α 0.785	Cron. α 0.734	Cron. α 0.637	Cron. α 0.928	Cron. α 0.928	Cron. α 0.893	Cron. α 0.785	Cron. α 0.734	Cron. α 0.637
1. I feel unrefreshed when I wake up in the morning	0.669	0.796				0.786	0.777	0.163			
2. My muscles feel stiff and achy	0.619	0.791				0.78	0.736	0.364			
3. I have anxiety attacks	0.460		0.684			0.636	0.626		0.219		
4. I grind or clench my teeth	0.391			0.608		0.493	0.436			0.585	
5. I have problems with diarrhea and/or constipation	0.428	0.549				0.54	0.591	−0.251			
6. I need help in performing my daily activities	0.477	0.666				0.655	0.618	0.315			
7. I am sensitive to bright lights	0.446			0.688		0.563	0.553			0.238	
8. I get tired very easily when I am physically active	0.737	0.859				0.848	0.805	0.35			
9. I feel pain all over my body	0.634	0.796				0.786	0.737	0.375			
10. I have headaches	0.353			0.624		0.513	0.494			0.308	
11. I feel discomfort in my bladder and/or burning when I urinate	0.383				0.647	0.541	0.526				0.389^a^
12. I do not sleep well	0.565	0.695				0.684	0.685	0.102^a^			
13. I have difficulty concentrating	0.728		0.861			0.795	0.772		0.487		
14. I have skin problems such as dryness, itchiness, or rashes	0.429	0.504				0.496	0.54	−0.229			
15. Stress makes my physical symptoms get worse	0.655		0.799			0.741	0.762		0.00822^a^		
16. I feel sad or depressed	0.563		0.735			0.684	0.668		0.301		
17. I have low energy	0.735	0.852				0.839	0.816	0.241			
18. I have muscle tension in my neck and shoulders	0,568	0.722				0.712	0.72	0.0584^a^			
19. I have pain in my jaw	0.434			0.741		0.605	0.552			0.656	
20. Certain smells, such as perfumes, make me feel dizzy and nauseated	0.418			0.647		0.536	0.523			0.255	
21. I have to urinate frequently	0.403				0.661	0.554	0.544				0.796^a^
22. My legs feel uncomfortable and restless when I am trying to go to sleep at night	0.472	0.605				0.593	0.586	0.142^a^			
23. I have difficulty remembering things	0.529		0.576			0.537	0.482		0.549		
24. I suffered trauma as a child	0.268		0.438			0.407	0.406		0.114^a^		
25. I have pain in my pelvic area	0.470				0.767	0.642	0.652				0.106^a^

*Abbreviations*: *EFA* exploratory factor analysis, *CFA* confirmatory factor analysis, *n* number of cases, *Cron. α* Cronbach’s alpha, *TLI* Tucker-Lewis Index, *RMSEA* root mean square error of approximation, *CI* confidence interval

Acceptable values for the factor model indicators:

Following Browne and Cudeck (1992) model fit was judged excellent for RMSEA< 0.05, good for RMSEA< 0.08, mediocre for RMSEA< 0.1, and poor for RMSEA> 0.1

Following Schermelleh-Engel et al. (2003) a model fit was judged acceptable for TLI > 0.95 and good for TLI ≥ 0.97

^a^The marked factor loadings were not significant

#### Confirmatory factor analysis

A). Original 4-factor model proposed by Mayer et al. [17]: The model fit was good, with a TLI of 0.99, a RMSEA of 0.06 (90%-CI: [0.05; 0.07]), and *x*^2^(269) = 553.09, *p* < 0.001. As expected, all items showed significant positive factor loadings, with standardized coefficients ranging from 0.438 to 0.861. As demonstrated in Table 4, there were also significant positive correlations among all four factors (physical symptoms, emotional distress, headache, urological symptoms), indicating that individuals who showed high scores in one dimension were also likely to demonstrate high scores in the other dimensions.

**Table 4:** Inter-Factor correlations of the CFA of the 4-factor model

Factor 1	Factor 2	Correlation
physical symptoms	emotional distress	0.87
physical symptoms	headache	0.83
physical symptoms	urological symptoms	0.84
emotional distress	headache	0.74
emotional distress	urological symptoms	0.69
headache	urological symptoms	0.61

Significance for all listed inter-factor correlation was ≤0.001

B). The 1-factor model proposed by Cuesta-Vargas et al. [49]: The model fit was good, with a TLI of 0.98, a RMSEA of 0.08 (90%-CI: [0.07; 0.08]), and χ2(275) = 756.39, *p* < 0.001. All items showed significant positive factor loadings, with standardized coefficients ranging from 0.407 to 0.848.

C). The Bifactor model proposed by Cuesta-Vargas et al. [19]: The model fit was excellent, with a TLI of 0.99, a RMSEA of 0.05 (90%-CI: [0.04; 0.06]), and *x*^2^(250) = 430.6, *p* < 0.001. All items showed significant positive factor loadings for the general factor, but not all factor loadings were significant or positive for the other four latent factors, with standardized coefficients ranging from − 0.251 to 0.816.

Comparing the different models used in the CFA, the bifactor model fit the data significantly better (*x*^2^(19) = 115.1, *p* < .001) than the original 4-factor model, while the original 4-factor model fit the data significantly better than the 1-factor model (*x*^2^(6) = 136.9, *p* < .001).

#### Construct validity – discriminative power

The Kruskal-Wallis-Test indicated the presence of significant differences (*p* < 0.001) in CSI-GE total scores among the subject subgroups. Post-hoc analyses found that the control group scored lower than all the other groups except patients with only regional pain. Patients with multisite or FMS-related pain were not significantly different from each other but scored higher than the other groups (Table 5).

**Table 5:** Results of the Dunn-Bonferroni post-hoc test – pairwise comparisons of the groups

Sample 1 – Sample2	Std. Test Statistic	Adjusted Significance
Significant pairwise comparisons		
HC - RAR	−5.13	< 0.001
HC - CBNP	−7.49	< 0.001
HC - MCP	−10.14	< 0.001
HC - FMS	−10.2	< 0.001
RCP - FMS	4.69	< 0.001
RCP - MCP	3.9	0.001
RAR - MCP	4.24	< 0.001
RAR - FMS	5.11	< 0.001
CBNP - FMS	4.36	< 0.001
CBNP - MCP	3.32	0.013
Not significant pairwise comparisons		
HC - RCP	−2.71	0.101
RCP - CBNP	−1.92	0.827
RCP - RAR	−0.88	1.0
RAR - CBNP	1.43	1.0
MCP - FMS	1.48	1.0

Each row tests the null hypothesis that the Sample 1 and Sample 2 distributions are the same. Asymptotic significances (2-sided tests) are displayed. The significance level is .05. Significance values have been adjusted by the Bonferroni correction for multiple tests (adjusted significance)

*Abbreviations*: *HC* healthy controls, *FMS* fibromyalgia syndrome, *MCP* multisite chronic pain, *CBNP* chronic back and neck pain, *RAR* rheumatoid arthritis in status of remission, *RCP* regional chronic pain

#### Construct validity - correlations

Using the adapted figures for Kendall’s τ for the classification, the CSI-GE demonstrated medium correlations with the PHQ-15 (τ = 0.57) and FSQ-SSS (τ = 0.56), and low correlations with the FSQ-WPI (τ = 0.47), CSI-GE-Part-B (τ = 0.45), DASS-Anxiety (τ = 0.45), DASS-Stress (τ = 0.43), PainDETECT-subscore (τ = 0.43), DASS-Depression (τ = 0.41), VonKorff (τ = 0.36), VR12-MCS (τ = − 0.35), MPSS (τ = 0.32), general well-being (τ = − 0.28), PCS (τ = 0.28), pain intensity (τ = 0.27), PSQm (τ = 0.23) and negligible or no significant correlations with pain duration and VR12-PCS (Supplement 4).

#### Construct validity – hypotheses testing

The results of the hypotheses testing approach can be observed within Table 6.

**Table 6:** Construct validity – Hypotheses testing

**Hypothesis- Group Comparison**	**Expected Outcome**	**Results**	**Was the Hypothesis met?**
CSI score of the HC group	HC group scoring the lowest	HC group showed lowest mean CSI-GE score, significantly different from all but the RCP group	Yes
CSI score of the FMS group	FMS group scoring the highest of all pain groups	highest mean CSI-GE score, significantly different from all but the MCP group	Yes
CSI score of the MCP group	MCP group scoring high in comparison to other pain groups but probably lower than the FMS group	Second highest mean CSI-GE score of all 5 pain groups	Yes
CSI score of the RCP group	RCP group scoring low in comparison to other pain groups	Lowest mean CSI-GE score of all 5 pain groups but higher than HC group (although not significant)	Yes
CSI Score of the RAR group	RAR group Scoring lower in comparison to FMS and MCP	Lower mean CSI–GE score than in FMS and in MCP groups	Yes
**Questionnaire- Correlations with CSI-GE Score**	**Expected Outcome**	**Kendal’s τ, the associated Holm adjusted*p*value and classification**	**Was the Hypothesis met?**
CS is related to subjective pain sensitivity	High correlation between CSI-GE and PSQminor	τ = 0.23; *p* < 0.001; low	No
CS is related to somatization or general symptom load	At least medium correlation between CSI-GE and (a) PHQ15 and (b) FSQ subscale SSS	a: τ = 0.57; *p* < 0.001; medium b: τ = 0.56; *p* < 0.001; medium	Yes
CS is related to spread of pain	At least medium correlation between CSI-GE and FSQ subscale WPI	τ = 0.47; *p* < 0.001; low	No
CS is related to neuropathic pain characteristics	At least medium correlation between CSI-GE and PainDETECT	τ = 0.43; *p* < 0.001; low	No
CS is related to chronification of pain	At least medium correlation between CSI-GE and MPSS	τ = 0.32; *p* < 0.001; low	No
CS is related to pain catastrophizing	At least medium correlation between CSI-GE and PCS	τ = 0.28; *p* < 0.001; low	No
**How many Hypotheses were met?**			**6/11 (55%)**

*Abbreviations*: *CS* central sensitization, *HC* healthy control, *FMS* Fibromyalgia, *MCP* multisite chronic pain, *RCP* reginal chronic pain, *RAR* Rheumatoid arthritis in remission, *PHQ15* Patient Health Questionnaire 15, *FSQ-SSS* Fibromyalgia Survey Questionnaire-Somatic Severity Score, *FSQ-WPI* Fibromyalgia Survey Questionnaire-Widespread Pain Index, *DASS* Depression Anxiety Stress Scale, *PCS* Pain Catastrophising Scale, *MPSS* Mainz Pain Staging System, *PSQm* Pain Sensitivity Questionnaire minor

## Discussion

The objective of this study was to create a culturally-adapted German version of the CSI and to test its psychometric properties. Internal consistency of different CSI translations has been examined in most international studies (Supplement 1) using Cronbach’s α, which has ranged from 0.87 [58, 59] to 0.993 [60], and is in agreement with our result of a Cronbach’s α of 0.928. Test-retest reliability has been examined using Pearson’s correlation, with r_p_ = 0.817 [17] and intra-class correlation, with results ranging from 0.85 [61] to 0.991 [60], which was in line with the ICC_2,1_ of 0.917 in our analysis. SEM and SDC were computed only by a few of the initial validation studies. The standard error of measurement (SEM) ranged from 0.31 [59] to 3.16 [62], whereas the smallest detectable change (SDC), which is also called minimal detectable change (MDC), ranged from 0.86 [59] to 8.12 [62]. Therefore, the values of SEM = 4.144 and SDC = ± 11.486 in our study seem high. However, the time interval with a mean of 18.42 days between the two measurement time points was longer than in the other studies. This longer interval may have reduced memory effects but may also represent relevant fluctuations in symptoms over the longer (baseline) observation interval since all patients reporting relevant changes in health prior to the retest were excluded.

A 4-factor structure of the CSI was originally determined by Mayer et al. [17] and supported by further international studies [10, 62, 63]. Other studies have demonstrated a 1-factor [49, 58] or 5-factor structure [61]. Due to the diverse reports of the factor structure of different CSI translations, Cuesta-Vargas et al. [19] performed a large FA with pooled data from multiple countries and multiple language-versions of the CSI. They demonstrated that the best fit was a bifactor model, with one general factor of “CS-related symptoms” and four latent factors. Considering that the bifactor model provided the best fit in our CFA and the EFA yielded a 1-factor solution, it seems justified to only compute an overall sum score for the CSI-GE, representing one universal general construct underlying all items. The four remaining latent factors within the bifactor-model suggest an underlying structure of specific factors that enclose these specific features. Our analysis does not support the use of subscales with the CSI-GE due to the non-significant or low loadings of the four latent factors (Table 3) and the questionable additional benefit of subscales. These findings are in line with Cuesta-Vargas et al. [19] who recommended that only total CSI scores be used and reported.

Assessing construct validity with the hypotheses testing approach only 55% of the hypotheses were met, which can not be considered an entirely persuasive and satisfactory result. However, the construct of the CSI-GE showed convincing evidence regarding the group comparisons and overall symptom load (PHQ, SSS), but demonstrated limited construct validity with respect to hypotheses more closely related to the construct of central sensitisation itself. Nevertheless, only the PHQ and SSS can be considered comparator instruments, the other questionnaires measured potentially related yet different constructs than the CSI. Subsequently, individual hypotheses, as well as the explorative correlations are further discussed.

The CSI-GE construct was supported by its ability to differentiate between subgroups believed to have different degrees of CS. The FMS and MCP groups scored significantly higher than all other groups except each other. This was expected as those groups were believed most likely to have the highest degree of CS. Also, the HC group differed significantly from all pain groups except the RCP group, which was believed to most likely have lower levels of CS. These finding are supported by Knezevic et al. [64], who found similar results in FMS, multiple pain sites, and localized pain subgroups.

However, correlations with other instruments were not as unequivocal. The highest correlations with somatic symptom severity may partly be explained by overlapping items between instruments. FMS patients, known to score highly on these instruments and to embody features of CS [65], were the highest scoring group in our study and indicate that symptom load is a key aspect of the CSI-GE construct. This symptom load may otherwise simply reflect the degree of polysymptomatic distress as suggested by Wolfe et al. for FMS [66] and RA [67].

All three scales of the DASS showed a low, positive correlation with the CSI-GE, demonstrating an increasing negative affective/emotional burden with increasing scores in the CSI-GE. Chiarotto et al. [58] showed positive correlations with depression and anxiety measures with the Italian version of the CSI as well.

The correlation between the CSI-GE and the painDETECT showed a small association with neuropathic pain components. Rehm et al. [35] found that the painDETECT indicated possible neuropathic pain in more than 50% of FMS patients. It is unclear whether this hints at neuropathic pain components in FMS patients, the presence of CS, or simply increased somatic symptoms. It is unlikely that neuropathic pain was a primary symptom in our patient sample since we attempted to exclude patients with a primary neuropathic pain component. The correlation between the painDETECT and the CSI-GE may suggest that either CS-related symptoms overlap with neuropathic pain features, or that the CSI truly represents symptoms related to CS and that the painDETECT results in false-positive detection of neuropathic pain in the presence of CS. This second assumption has been supported by a qualitative study [68].

The degree of chronification (MPSS) and severity (Korff) of chronic pain both correlated poorly with the CSI-GE. The slightly higher correlation with the Von Korff scale is possibly explained with pain-related disability, which is considered within the score. This is supported by findings of Kregel et al. [69] who showed positive correlations between the CSI and impairment caused by pain in daily life.

The PCS correlated to a small degree with the CSI-GE. This was unexpected as we believed that the concept of catastrophizing is an amplifying factor for CS, and previous CSI studies have reported higher correlations with the PCS [60, 64]. Correlations with pain sensitivity and intensity were low. The latter was also found by Knezevic et al. [64]. This appears somewhat counterintuitive, as CS is believed to trigger higher pain intensity and decreased thresholds to painful stimulation by increasing the sensitivity of the somatosensory system. Therefore, we expected a higher correlation with the PSQm, which captures individual sensitivity towards pain. In line with the low correlation with the PSQm, Kregel et al. [69] reported weak correlations between CSI total scores and pressure pain thresholds (PPT). Also Hendriks et al. [70] found that the CSI did not correlate significantly with PPT and concluded that the CSI captures the psychopathology associated with CS and not neurobiological alterations.

The CSI-GE demonstrated no association with the duration of pain. This finding is in line with other CSI studies [59, 61]. One might argue that with a longer chronic pain state, CS is expected to increase due to a higher degree of afferent input sensitizing the nervous system. On the other hand, CS could be understood as a self-maintaining process once initiated and not influenced by duration.

Our results support the negative correlations between mental quality of life and the CSI scores that have been found in previous studies [61, 64, 69]. Surprisingly in our study, the physical quality of life did not correlate significantly with the CSI-GE. This is in contrast to other validation studies showing significant negative correlations with the physical quality of life [58, 64, 69]. Considering that the CSI-GE correlated highest with instruments measuring the degree of somatic symptom load, a negative correlation with physical quality of life had been expected. However, Knezevic et al. [64] found lower correlations with SF-36-PCS than with SF-36-MCS supporting the finding that the mental component seems more prominent.

After the responses on CSI part B were summed, the FMS group reported the highest number of CSS-related diagnoses (3.6) and the HC group reported the lowest number (0.3). These results are in line with previous studies that have assessed CSI part B [17, 63]. Though we found a positive correlation between total scores on part A and the summed responses of part B of the CSI-GE, the correlation was low, considering that part B lists typical CSSs and related disorders, and part A includes symptoms associated with CSSs. However, these results should be interpreted with caution, as patients reported difficulties answering the questions in part B, which asks for previous diagnoses made by a physician. Patients may have marked diagnoses based on their subjective opinion and understanding of the respective label. Also, the comparability of the criteria a diagnosis was based on is unclear. Nevertheless, as noted previously, CSI part B is not designed to be scored when used clinically. It only provides additional information to help identify when a patient’s symptom presentation may be related to CS or be indicative of a CSS.

### Limitations

As with all studies of this kind, the results are based on a limited sample of subjects within two regions of Germany, so the findings may not generalize to other populations. More women than men were recruited as in previous CSI validation studies [10, 58], which is in accordance with more women being affected by musculoskeletal pain syndromes [71]. Slight age and sex differences between the subgroups may have influenced the group comparisons. However, our analysis demonstrated a very weak relationship between CSI-GE scores and age. A significant age difference was observed in only in one subgroup comparison (HC – RAR).

As there was no formal a priori definition of the hypothesis, the selected cut–off points (like high or medium correlation) remain somewhat arbitrary and of limited validity. The possibility of treatment effects, prior to data collection, on CSI scores needs to be considered. This highlights a potential limitation in ours, as well as other CSI validation studies, as they include little information about previous treatments such as psychotherapy, interdisciplinary multimodal pain therapy, or drug therapies that could affect questionnaire responses [72, 73]. One previous study has demonstrated that the CSI can be responsive to treatment interventions, as CSI scores improved in a group of chronic spinal pain patients who completed a functional restoration program [74]. The comparison of our results with previous studies must be interpreted cautiously because different correlation coefficients have been used and Kendall’s τ tends to be smaller in magnitude than Spearman rho correlation coefficient [75]. Our study did not include measures like QST to quantify the patients’ sensitivity and pain status. This should be explored in future studies. Although the calculation of overall sum scores for the CSI-GE, other CSI versions, and other patient-reported outcome measures is well established, it should be acknowledged that the summation of ordinal measured items must be viewed critically and is a point of controversy in the scientific literature [76, 77].

## Conclusion

The CSI-GE demonstrated robust psychometric properties as well as solid reliability. Based on the results of our factor analysis, it thus seems justified to compute one overall sum score. Our construct validation assessment suggests that the questionnaire reflects a dimension that no other tool that we compared has captured in the same way, although high symptom load was a prominent overlapping feature. Some of the correlations were unexpected within the current understanding of CS. It remains uncertain to which degree the CSI-GE captures CS and quantifies its symptoms. Combining our findings with previously published research regarding the CSI, we can conclude that interpretation of the total CSI score is made more difficult because definitions of CS are diverse; symptoms are broad and overlapping in a variety of conditions and may indicate polysymptomatic distress; new concepts such as nociplastic or chronic primary pain may be insufficiently considered; and no gold standard exists for the clinical or experimental quantification of CS. Experimental studies and studies examining the responsiveness to interventions in well-characterized patient groups may help to better define the scope of the instrument.

In conclusion, we recommend using the CSI-GE in clinical practice only with caution and primarily as a screening for symptoms that may be related to CS. The concept of CS requires further clarification within a research context. There is currently no established clinical diagnostic or treatment pathway in case of a positive screening result using the CSI-GE.

## Supplementary Information

**Additional file 1: Supplement 1.** Different international CSI validation studies in different languages.

**Additional file 2: Supplement 2.** The German translation and cross-cultural adaptation of the Central Sensitization Inventory (CSI-GE).

**Additional file 3: Supplement 3.** CENTRAL SENSITIZATION INVENTORY German version (CSI-GE)_V_19.09.2020.

**Additional file 4: Supplement 4.** Visualized pairwise correlations between the CSI-GE part A sum score and each questionnaire.

## Data Availability

The datasets generated and analyzed during the current study are not publicly available as they are currently also used on a dissertation. After completion, they will be publicly available. Nevertheless, the datasets can also be preliminary available from the corresponding author on reasonable request.

## Abbreviations

CBNP: Chronic back and/or neck pain
CFA: Confirmatory factor analyses
CPP: Chronic pain patients
CSI: Central Sensitization Inventory
CSI-GE: German version of the Central Sensitization Inventory
CS: Central Sensitization
CSS: Central Sensitivity Syndromes
DASS: Depression Anxiety Stress Scale
EFA: Exploratory factor analysis
FA: Factor analysis
FMS: Fibromyalgia
FSQ: Fibromyalgia Survey Questionnaire
FSQ-WPI: Fibromyalgia Survey Questionnaire - Widespread Pain Index
FSQ-SSS: Fibromyalgia Survey Questionnaire - Somatic Severity Score
HC: Healthy control group
ICC: Intraclass-correlation-coefficient
MCP: Multisite chronic pain
MFHW: Marburger questionnaire on habitual well-being
MPSS: Mainz Pain Staging System
PCS: Pain Catastrophizing Scale
PHQ15: Patient Health Questionnaire 15
PPT: Pressure pain thresholds
PSQm: Pain Sensitivity Questionnaire minor
QST: Quantitative Sensory Testing
RAR: Rheumatoid arthritis in remission
RCP: Regional chronic pain
SDC: Smallest Detectable Change
SEM: Standard Error of Measurement
VonKorff Scale: Graded Chronic Pain Scale by VonKorff
VR12: Veterans Rand 12
VR12-PCS: Veterans Rand 12-physical composite summary
VR12-MCS: Veterans Rand 12-mental composite summary
