# Supplementary material for: Cross-cultural adaptation and validation of the German Central Sensitization Inventory (CSI-GE)
Source: BMC Musculoskelet Disord. 2021 Aug 18;22:708. doi: 10.1186/s12891-021-04481-5 (PMC8375049; doi:10.1186/s12891-021-04481-5)
Supplement: Supplementary file 1 — Additional file 1: Supplement 1. Different international CSI validation studies in different languages. [file 12891_2021_4481_MOESM1_ESM.docx]

**Cross-cultural adaptation and validation of the German Central Sensitization Inventory (CSI-GE)**

**Supplement 1: Different international CSI validation studies in different languages**

M Klute ^a^, M Laekeman ^b^, K Kuss ^c^, F Petzke ^a^, A Dieterich ^d^, A Leha ^e^, R Neblett ^f^, S Ehrhardt ^g^, J Ulma ^h^, A Schäfer ^i^

^a^ Pain Medicine, Department of Anaesthesiology, University Medical Center Göttingen, Germany

^b^ Physiological Psychology, Otto-Friedrich- University of Bamberg, Germany

^c^ Department of General Practice/Family Medicine, Philipps University Marburg, Germany

^d^ Physiotherapy, Faculty of Health, Safety, Society, Furtwangen University, Germany

^e^ Department of Medical Statistics, University Medical Center Göttingen, Germany

^f^ PRIDE Research Foundation, Dallas, Texas, USA

^g^ Faculty of Social Sciences, City University of Applied Sciences, Bremen, Germany

^h^ Clinic for Pain Medicine Bremen, Rotes-Kreuz-Krankenhaus Bremen, Germany

^i^ Faculty of Social Work and Health, University of Applied Science and Art, Hildesheim, Germany

| **Language** | **Publication** | **Methods** | | |
| --- | --- | --- | --- | --- |
| English | Mayer et al. 2012[1]  Study 1 | Study design | n=210 CPP / n=149 HC | |
|  |  | Reliability | IC | Cronbachs α=0.879 |
|  |  |  | Test-Retest | TI= 5 days; n= 149 (HC only); Pearsons r= 0.817 |
|  |  | Validity | Structural | Explorative Analysis  PCA + promax rotation, n=359 (CPP+HC)  -> 4-factor-structure explaining 53.4% of the variance  F1 (physical symptoms): 2, 6, 8, 9, 12, 17, 18, 22 (30.9%)  F2 (emotional distress): 3, 13, 15, 16, 23, 24 (7.2%)  F3 (headache/jaw symptoms): 4, 7, 10, 19, 20 (10.1%)  F4 (urological symptoms): 11, 21, 25 (5.2%)  Variables without sufficient loading on any factor (<0.4): 1, 5, 14 |
|  | Mayer et al. 2012[1]  Study 2 | Study design | n=105 CPP + 40HC | |
|  |  | Validity:  Hypothesis testing | Differences between 4 Groups (HC, FMS, CLBP, CWP) were analysed showing a significant difference between HC and the pain groups with FMS scoring the highest regarding the overall sum score of CSI part A. In addition, the number of diagnoses in part B also showed a significant difference between FM and HC. | |
| Dutch | Kregel et al. 2016[2] | Study design | n=368 CPP /n=49 HC | |
|  |  | Reliability | IC | Cronbachs α=0,91 |
|  |  |  | Test-Retest | TI= 21 days; n=36 CPP -> ICC= 0.88; n=45 HC -> ICC=0.91 |
|  |  | Validity | Structural | -EFA + promax rotation, n=180 CPP  -> 4-Factor-structure  F1 (general disability and physical symptoms): 2, 6, 8, 9, 17, 25  F2 (higher central sensitivity): 4, 7, 10, 13, 18, 19, 20  F3 (urological and dermatological symptoms): 11, 14, 21  F4 (emotional distress): 3, 12, 13, 15, 16, 17  variables without sufficient loading on any factor (<0,4): 1, 5, 22, 23, 24  -CFA n=188 CPP (Evaluating results of EFA, the 5 factors not loading sufficiently in the EFA were not included)  -> a second-order factor model was examined, representing an underlying general CS factor which could explain the intercorrelations between the other factors |
|  |  |  | Discriminative power | Using ANCOVA controlling for age the CPP obtained significant higher scores on all four factors |
|  | Kregel et al. 2018[3] | Study design | N=116 CPP (chronic spinal pain) | |
|  |  | Validity | Convergent | CSI correlations  -Pressure pain thresholds r_p_=-0.237 to -0.276  -Conditioned pain modulation (CPM) effects r_p_=0.017  -Current pain intensity r_s_=0.32 (NRS)  -Pain disability r_p_=0.472  -Pain catastrophizing r_p_=0.464 (PCS)  -To lower physical r_p_=-0.617 and mental r=-0.635 quality of life (SF36) |
| French | Pitance et al. 2016[4]  (only an abstract is available and therefore the information is very limited) | Study design | n=40 CPP (FMS) + n=40 Patients having a „sprained ankle“ + n=40 HC | |
|  |  | Reliability | IC | Not mentioned in the abstract |
|  |  |  | Test-Retest | TI= 7days; CPP ICC=0.94; HC ICC=0.91 |
|  |  | Validity | Structural | CFA based on EFA of Mayer et al.  (five alternative models were tested) |
|  |  |  | Convergent | Correlations to Pain Catastrophizing Scale, Hospital Anxiety and Depression Scale, Brief Pain Inventory, SF-36 |
|  |  |  | Discriminative power | Significant difference between CPP (FMS) and HC concerning the CSI score |
| Spanish | Cuesta-Vargas et al. 2016[5] | Study design | n=395 CPP (no HC included) | |
|  |  | Reliability | IC | Cronbachs α=0.872 |
|  |  |  | Test-Retest | TI= 7days; n= 45; ICC=0.91 (allowed error of +/- 10%) |
|  |  | Validity | Structural | Explorative Analysis  PCA + MLE, only CPP  -> 1-Factor-structure explaining 25.9% of the variance |
| Gujarati | Bid et al. 2016[6] | Study design | n=31 CPP (no HC included) | |
|  |  | Reliability | IC | Cronbachs α=0.914 |
|  |  |  | Test-Retest | TI= 7days; n=31; ICC=0.971 |
|  |  | Validity | Content | A 23 members expert panel rated on a 7-point Likert Scale the equivalence to the English original as well as the relevance and representativeness of the instrument assessing CS |
| Portuguese (Brazilian) | Caumo et al. 2017[7] | Study design | n=222 CPP + n=63 HC | |
|  |  | Reliability | IC | Cronbachs α=0.91 |
|  |  |  | Test-Retest | TI= 15days; n=20 (female patients with FMS); ICC= 0.91 |
|  |  | Validity | Structural | Factor Analysis  PCA + promax rotation n=285 (CPP+HC)  -> 4-Factor-structure explaining 49.1% of the variance supporting the English original structure  F1 (physical Symptoms): 2, 6, 8, 9, 12, 17, 22 (32.81%)  F2 (Emotional Distress): 1, 3, 7, 13, 15, 16, 23, 24 (6.09%)  F3 (Headache/Jaw Symptoms): 4, 10, 18,19, 20 (5.36%)  F4 (Urological Symptoms): 5, 11, 21, 25 (4.94%)  Variables without sufficient loading on any factor (<0.4): 14 |
|  |  |  | Discriminative power | -Ability of the CSI Score to differ between CS symptom groups  -Ability of the CSI Score to differ between chronic pain conditions |
|  |  |  | Convergent | Correlation between Pain Catastrophizing Scale and the CSI Score was found to be moderate |
|  |  |  | Content | In a subsample (66 CPP + 11 HC) CSI scores were correlated with two markers for CS (CPM task and BDNF (Brain-derived neurotropic factor) blood sample  -Increase in BDNF was associated with higher CSI scores r_p_=0.52  -Conditioned Pain Modulation (CPM) nonresponders had significantly higher CSI scores |
| Japanese | Tanaka et al. 2017[8] | Study design | n=290 CPP (no HC included) | |
|  |  | Reliability | IC | Cronbachs α=0.89 |
|  |  |  | Test-Retest | TI= 7days; n=158; ICC=0.85 |
|  |  | Validity | Structural | EFA using MLE + promax rotation  -> 5-Factor-structure  F1 (Emotional distress): 15, 16, 17, 24  F2 (Urological and general Symptoms): 9, 11, 21, 22, 23, 25  F3 (Muscle Syptomes): 2, 18  F4 Headache/Jaw): 4, 10, 19  F5 (sleep disturbance): 1, 8, 12  Variables without sufficient loading on any factor (<0.4): 3, 5, 6, 7, 13, 14, 20 |
|  |  |  | Convergent | Relationship to:  -EuroQol 5-dimensions (health related Quality of life) r_s_=-0.44  -Brief Pain Inventory containing pain intensity r_s_=0.42 and pain interference r_s_=0.48, non to the duration of pain r_s_=0.1 |
| Serbian | Knezevic et al. 2018[9] | Study design | n=355 CPP + n=34 HC | |
|  |  | Reliability | IC | Cronbachs α=0.909 |
|  |  |  | Test-Retest | TI= 7days (+/-1); n=137; ICC= 0.947 |
|  |  | Validity | Structural | CFA was used testing the following models:  -original 4-Factor Model by Mayer et al.  -1-factor model by Cuesta-Vargas et al.  -the possibility of a hierarchical single second order factor was tested  ->the 4-Factor model provided the best fit but the presence of a single second order factor was also detected |
|  |  |  | Convergent | Analysis of the severity Groups proposed by Neblett et al. 2016 ->Significant differences in severity and duration of pain in the severity groups |
|  |  |  | Discriminative power | CSI showed significant differences in all three subject groups (FMS, localized, HC) |
|  | Knezevic et al. 2020[10] | Study design | n=146 HC + n=399 CPP (155 low back pain, 26 cervical pain, 46 localized extremity pain, 20 temporomandibular pain, 5 lateral epicondylitis, 5 complex regional pain syndrome (CRPS), 47 fibromyalgia, 95 pain in more than 1 location) | |
|  |  | Validity | Convergent | Correlations between CSI score and other Patient-Reported questionnaires using Kendall´s Tau and Bonferroni correction for multiple comparisons  Current pain intensity τ=0.271/ strongest pain intensity (past 4 weeks) τ=0.258 / average pain intensity (past 4 weeks) τ=0.277 (numeric rating scales)  Fear-Avoidance Components Scale (FACS) total score τ=0.381  Pain Catastrophising Scale (PCS) total score τ=0.369  Oswestry Disability Index (ODI) τ=0.381  Multidimensional Scale of Perceived Social Support (MSPSS) total score τ=-0.186  Medical Outcomes Study (MOS) cognitive functioning scale τ=-0.409 / MOS sleep scale τ=-0.504  Short Form-36 (SF-36) quality-of-life physical composite summary (PCS) τ=-0.292 / mental composite summary MCS τ=-0.402 |
|  |  |  | Discriminative power | -Significant difference in mean total CSI scores between 6 patient groups (grouping by pain diagnosis) and the HC group were found. Lowest scores in the HC group and highest in the FMS group followed by the multiple pathologies subgroup.  -Using the CSI total score CSI severity groups were assembled. The difference between the different severity groups for each Patient-Reported questionnaire was analysed. Overall most variables increased with the CSI severity level. |
| Italian | Chiarotto et al. 2018[11] | Study design | n=220 CPP (no HC included) | |
|  |  | Reliability | IC | Cronbachs α=0.87 |
|  |  |  | Test-Retest | not assessed |
|  |  | Validity | Structural | Explorative Analysis  MLE + promax rotation using kaiser normalisation  -> 1-Factor-structure explaining 26% of the variance  21 of the 25 variables loaded on that factor below 0.4 |
|  |  |  | Construct | Hypothesis testing (10 Hypothesis)  8/10 Hypothesis were met showing satisfactory construct validity  -mean difference >10 points in CSI score between FMS and no-FMS groups  -mean increase of 4,5 points in CSI score for every additional diagnosis in part B  Correlations to CSI Score:  -Pain intensity (Numeric Rating scale) 0.427  -Physical functioning (10 item SF-36-PF) -0.479  -Anxiety 0.706 and depression 0.551 (Hospital Anxiety and Depression Scale) |
| Polish | Turczyn et al. 2019[12] | The CSI was translated and culturally adapted in to Polish. It is now ready for validation and can be introduced to clinical practice | | |
| Greek | Bilika et al. 2020[13] | Study design | n=200 CPP + 50 HC | |
|  |  | Reliability | IC | Cronbachs α= 0.993 |
|  |  |  | Test-Retest | TI= 5 to 7 days  n= 30 (CPP)  ICC=0.991 |
|  |  | Validity | Structural | Not assessed |
|  |  |  | Discriminative Power | -Significant differences between four subgroups (FMS, single body area, multiple pain complaints, HC) were observed (except between single and multiple pain groups)  -FMS scoring the highest and HC the lowest  -Significant differences were also found between the 5 CSI severity subgroups |
|  |  |  | Convergent | Correlation to Pain Catastrophizing Scale r_s_=0.68 |
| Russian | Esin et al. 2020[14] | Study design | In English only an abstract on PubMed is available and therefore the information is very limited. Furthermore, no concrete figures are mentioned within the abstract. | |
| Nepali | Sharma et al. 2020[15] | Study design | n=100 (sub-acute and chronic musculoskeletal pain) | |
|  |  | Reliability | IC | Cronbachs α= 0.87 |
|  |  |  | Test-Retest | TI= 2 weeks; ICC= 0.98 |
|  |  | Validity | Construct | Five Hypothesis were tested (4/5 were met) using Spearman correlations between the CSI score and other instruments  -Pain Catastrophising Scale (PCS) r_s_=0.5  -Total number of pain descriptors r_s_=0.35  -Pain intensity (NRS) r_s_=0.25  -The mean CSI Score was significantly higher in women  -Pain duration r_s_=0.1 (not significant) |
| Pooled Multicounty Sample | Cuesta-Vargas et al. 2018[16] | Study design | n=2093 including n=192 nonclinical subjects; subjects included into the analysis n=1987, subjects excluded n=109  (subject data reported in this multicountry had previously been published in individual CSI translation studies) | |
|  |  | Reliability | IC | Cronbachs α =0.92 |
|  |  | Validity | Structural | Random division into 2 subsamples for cross-sample validation  **PCA** (subsample n=1049)  -4 factors with eigenvalues >1 (ratio between the 1. and 2. Factor and scree plot suggested a 1 factor solution  -1-factor model explaining 36.1% of the variance  **CFA** (subsample n=1044)  -1-factor model: CFI = .91 and TLI = 0.90 and RMSEA = .08 (95% CI, 0.08–0.09)  -Original 4-factor model (CFI = 0.93, TLI = 0.93, RMSEA = 0.07, 95% CI = 0.07–0.08) It fit the data better than the 1-factor model  -Bifactor model including 1 global “CS-related symptoms” and 4 specific latent factors (4-factor structure of original 4-factor model) showed the best fit across all indices (CFI = 0.96, TLI = 0.95, RMSEA = 0.06, 95% CI, 0.06–0.07). The bifactor model resulted in a significantly better fit to the data than the 1-factor model (Δχ^2^ test = 881.067, df = 25, P < 0.0001, ΔTLI = 0.05). For the general factor that includes all items, the factor loadings ranged from 0,39 to 0,77 |
| Abbreviations:  CPP- chronic pain patients, HC- healthy controls, IC- Internal consistency, TI- time-interval, r_p_ - Pearson correlation coefficient, r_s_ - Spearman’s correlation coefficient, FA- Factor Analysis, PCA- principal component analysis, EFA- exploratory FA, CFA- confirmatory FA, MLE- Maximum Likelihood Extraction, ICC- Intraclass Correlation Coefficient (different types were used), FMS- Fibromyalgia, CWP- chronic widespread pain patients, CLBP- chronic regional lumbar pain patients | | | | |

**Bibliography**

1. Mayer TG, Neblett R, Cohen H, Howard KJ, Choi YH, Williams MJ, et al. The Development and Psychometric Validation of the Central Sensitization Inventory. Pain Pract. 2012;12:276–85.

2. Kregel J, Vuijk PJ, Descheemaeker F, Keizer D, van der Noord R, Nijs J, et al. The Dutch Central Sensitization Inventory (CSI): Factor Analysis, Discriminative Power, and Test-Retest Reliability. Clin J Pain. 2016;32:624–30.

3. Kregel J, Schumacher C, Dolphens M, Malfliet A, Goubert D, Lenoir D, et al. Convergent Validity of the Dutch Central Sensitization Inventory: Associations with Psychophysical Pain Measures, Quality of Life, Disability, and Pain Cognitions in Patients with Chronic Spinal Pain. Pain Pract. 2018;18:777–87.

4. Pitance L, Piraux E, Lannoy B, Meeus M, Berquin A, Eeckhout C, et al. Cross cultural adaptation, reliability and validity of the French version of the central sensitization inventory. Manual Therapy. 2016;25:e83–4.

5. Cuesta-Vargas AI, Roldan-Jimenez C, Neblett R, Gatchel RJ. Cross-cultural adaptation and validity of the Spanish central sensitization inventory. Springerplus. 2016;5:1837.

6. Bid DD, Soni NC, Rathod PV, Ramalingam AT. Content Validity and Test-Retest Reliability of the Gujarati Version of the Central Sensitization Inventory. Natl J Integr Res Med. 2016;7:18–24.

7. Caumo W, Antunes LC, Elkfury JL, Herbstrith EG, Busanello Sipmann R, Souza A, et al. The Central Sensitization Inventory validated and adapted for a Brazilian population: psychometric properties and its relationship with brain-derived neurotrophic factor. J Pain Res. 2017;10:2109–22.

8. Tanaka K, Nishigami T, Mibu A, Manfuku M, Yono S, Shinohara Y, et al. Validation of the Japanese version of the Central Sensitization Inventory in patients with musculoskeletal disorders. PLoS One. 2017;12:e0188719.

9. Knezevic A, Neblett R, Jeremic‐Knezevic M, Tomasevic‐Todorovic S, Boskovic K, Colovic P, et al. Cross-Cultural Adaptation and Psychometric Validation of the Serbian Version of the Central Sensitization Inventory. Pain Pract. 2018;18:463–72.

10. Knezevic A, Neblett R, Colovic P, Jeremic-Knezevic M, Bugarski-Ignjatovic V, Klasnja A, et al. Convergent and Discriminant Validity of the Serbian Version of the Central Sensitization Inventory. Pain Pract. 2020;20:724–36.

11. Chiarotto A, Viti C, Sulli A, Cutolo M, Testa M, Piscitelli D. Cross-cultural adaptation and validity of the Italian version of the Central Sensitization Inventory. Musculoskelet Sci Pract. 2018;37:20–8.

12. Turczyn P, Kosińska B, Janikowska-Hołoweńko D, Malec-Milewska M, Marszalec N, Maleszka P, et al. Translation and cross-cultural adaptation of the Polish Central Sensitization Inventory. Reumatologia/Rheumatology. 2019;57:129–34.

13. Bilika P, Neblett R, Georgoudis G, Dimitriadis Z, Fandridis E, Strimpakos N, et al. Cross-cultural Adaptation and Psychometric Properties of the Greek Version of the Central Sensitization Inventory. Pain Pract. 2020;20:188–96.

14. Esin OR, Gorobets EA, Khairullin IK, Esin RG, Gamirova RG, Shamsutdinova RF, et al. [Central Sensitization Inventory - a Russian version]. Zh Nevrol Psikhiatr Im S S Korsakova. 2020;120:51–6.

15. Sharma S, Jha J, Pathak A, Neblett R. Translation, cross-cultural adaptation, and measurement properties of the Nepali version of the central sensitization inventory (CSI). BMC Neurol. 2020;20:286.

16. Cuesta-Vargas AI, Neblett R, Chiarotto A, Kregel J, Nijs J, van Wilgen CP, et al. Dimensionality and Reliability of the Central Sensitization Inventory in a Pooled Multicountry Sample. J Pain. 2018;19:317–29.
