# Supplementary material for: Cross-cultural adaptation and validation of the German Central Sensitization Inventory (CSI-GE)
Source: BMC Musculoskelet Disord. 2021 Aug 18;22:708. doi: 10.1186/s12891-021-04481-5 (PMC8375049; doi:10.1186/s12891-021-04481-5)
Supplement: Supplementary file 2 — Additional file 2: Supplement 2. The German translation and cross-cultural adaptation of the Central Sensitization Inventory (CSI-GE). [file 12891_2021_4481_MOESM2_ESM.docx]

**Cross-cultural adaptation and validation of the German Central Sensitization Inventory (CSI-GE)**

**Supplement 2: The German translation and cross-cultural adaptation of the Central Sensitization Inventory (CSI-GE)**

M Klute ^a^, M Laekeman ^b^, K Kuss ^c^, F Petzke ^a^, A Dieterich ^d^, A Leha ^e^, R Neblett ^f^, S Ehrhardt ^g^, J Ulma ^h^, A Schäfer ^i^

^a^ Pain Medicine, Department of Anaesthesiology, University Medical Center Göttingen, Germany

^b^ Physiological Psychology, Otto-Friedrich- University of Bamberg, Germany

^c^ Department of General Practice/Family Medicine, Philipps University Marburg, Germany

^d^ Physiotherapy, Faculty of Health, Safety, Society, Furtwangen University, Germany

^e^ Department of Medical Statistics, University Medical Center Göttingen, Germany

^f^ PRIDE Research Foundation, Dallas, Texas, USA

^g^ Faculty of Social Sciences, City University of Applied Sciences, Bremen, Germany

^h^ Clinic for Pain Medicine Bremen, Rotes-Kreuz-Krankenhaus Bremen, Germany

^i^ Faculty of Social Work and Health, University of Applied Science and Art, Hildesheim, Germany

Different translation methods for questionnaires are recommended without evidence in favour of one specific method; a multistep approach has been advised to increase the quality [1]. The cross-cultural adaptation of the English CSI into German was conducted following the recommendations of the guideline of the American Association of Orthopaedic Surgeons Outcomes Committee [2]. This guideline recommends five steps for the cross-cultural adaptation of self-report measures:

1) forward translation

2) synthesis

3) backward translation

4) expert committee review

5) pretest

Step 1: Two German native speakers with excellent English language skills translated the English CSI into German independently from each other. One translator was a physiotherapist and familiar with pain-related questionnaires, the other was a naive translator not familiar with the CSI concept and without medical background.

Step 2: The results of the two different forward translations were discussed with the translators and the expert committee to develop a synthesis version. The expert committee included six experts (e.g. health, research and language professionals).

Step 3: Two yet not involved native English speakers with excellent knowledge of German independently translated the German synthesis version back into English. Both did not know the original English version of the CSI. Here, one of the translators was a physiotherapist and the second translator worked as an expert for occupational safety and health.

Step 4: The translation team and the expert committee, advised by an author of the original CSI (R.N.), discussed and finally agreed upon a pre-final synthesis version for the pretest.

Step 5: In the pretest, 15 volunteers with chronic pain, recruited at the Pain Clinic of the University Medical Center Göttingen, completed the prefinal version and were interviewed to assess the comprehensibility, the usability and the acceptance of the prefinal version. The Three-Step Test-Interview was conducted as a pretest procedure. This method combines different cognitive survey techniques in three consecutive steps. For evaluation, the interviews were recorded with an audio recorder and then evaluated for content analysis. The pretest resulted in some suggestions for improvements. The experts discussed the suggestions and adapted two items. The resulting CSI-GE was tested for its psychometric properties in the validation study.

The steps of the translation and the cross-cultural adaptation of the CSI-GE have been published elsewhere [3].

**Bibliography:**

1. Acquadro C, Conway K, Hareendran A, Aaronson N. Literature Review of Methods to Translate Health-Related Quality of Life Questionnaires for Use in Multinational Clinical Trials. Value in Health. 2008;11:509–21.

2. Beaton DE, Bombardier C, Guillemin F, Ferraz MB. Guidelines for the Process of Cross-Cultural Adaptation of Self-Report Measures. Spine (Phila Pa 1976). 2000;25:3186–91.

3. Laekeman M, Kuss K, Seeger D, Schäfer A. Zentrale Sensibilisierung erkennen. Der Central Sensitization Inventory wird ins Deutsche übersetzt und validiert. pt Zeitschrift für Physiotherapeuten. 2017;:71–3.
