# Supplementary material for: Cross-cultural adaptation and validation of the German Central Sensitization Inventory (CSI-GE)
Source: BMC Musculoskelet Disord. 2021 Aug 18;22:708. doi: 10.1186/s12891-021-04481-5 (PMC8375049; doi:10.1186/s12891-021-04481-5)
Supplement: Supplementary file 3 — Additional file 3: Supplement 3. CENTRAL SENSITIZATION INVENTORY German version (CSI-GE)_V_19.09.2020. [file 12891_2021_4481_MOESM3_ESM.docx]

**Cross-cultural adaptation and validation of the German Central Sensitization Inventory (CSI-GE)**

**Supplement 3: CENTRAL SENSITIZATION INVENTORY German version (CSI-GE)**

M Klute ^a^, M Laekeman ^b^, K Kuss ^c^, F Petzke ^a^, A Dieterich ^d^, A Leha ^e^, R Neblett ^f^, S Ehrhardt ^g^,

J Ulma ^h^, A Schäfer ^i^

^a^ Pain Medicine, Department of Anaesthesiology, University Medical Center Göttingen, Germany

^b^ Physiological Psychology, Otto-Friedrich- University of Bamberg, Germany

^c^ Department of General Practice/Family Medicine, Philipps University Marburg, Germany

^d^ Physiotherapy, Faculty of Health, Safety, Society, Furtwangen University, Germany

^e^ Department of Medical Statistics, University Medical Center Göttingen, Germany

^f^ PRIDE Research Foundation, Dallas, Texas, USA

^g^ Faculty of Social Sciences, City University of Applied Sciences, Bremen, Germany

^h^ Clinic for Pain Medicine Bremen, Rotes-Kreuz-Krankenhaus Bremen, Germany

^i^ Faculty of Social Work and Health, University of Applied Science and Art, Hildesheim, Germany

| **CENTRAL SENSITIZATION INVENTORY German version (CSI-GE)****„Zentraler Sensibilisierungsfragebogen“ TEIL A** |
| --- |

### **Bitte umkreisen Sie bei jeder Aussage die für Sie aktuell am besten passende Antwort auf der rechten Seite.**

| 1. Wenn ich morgens aufwache, fühle ich mich müde  und nicht erholt. | nie | selten | gelegentlich | | oft | immer |
| --- | --- | --- | --- | --- | --- | --- |
| 2. Meine Muskeln fühlen sich steif und schmerzhaft an. | nie | selten | gelegentlich | | oft | immer |
| 3. Ich habe Angstattacken. | nie | selten | gelegentlich | | oft | immer |
| 4. Ich knirsche oder beiße meine Zähne zusammen. | nie | selten | gelegentlich | | oft | immer |
| 5. Ich habe Probleme mit Durchfall und/oder  Verstopfung. | nie | selten | gelegentlich | | oft | immer |
| 6. Ich brauche Hilfe bei der Verrichtung meiner  Alltagstätigkeiten. | nie | selten | gelegentlich | | oft | immer |
| 7. Ich reagiere empfindlich auf helles Licht. | nie | selten | gelegentlich | | oft | immer |
| 8. Ich ermüde sehr schnell bei körperlichen Aktivitäten. | nie | selten | gelegentlich | | oft | immer |
| 9. Ich habe am ganzen Körper Schmerzen. | nie | selten | gelegentlich | | oft | immer |
| 10. Ich habe Kopfschmerzen**.** | nie | selten | gelegentlich | | oft | immer |
| 11. Meine Blase fühlt sich unangenehm an und/oder ich  habe Brennen beim Wasserlassen. | nie | selten | gelegentlich | | oft | immer |
| 12. Ich schlafe nicht gut. | nie | selten | gelegentlich | | oft | immer |
| 13. Ich habe Konzentrationsschwierigkeiten. | nie | selten | gelegentlich | | oft | immer |
| 14. Ich habe Hautprobleme, wie z.B. trockene oder  juckende Haut oder Hautausschlag. | nie | selten | gelegentlich | | oft | immer |
| 15. Stress verstärkt meine körperlichen Beschwerden. | nie | selten | gelegentlich | | oft | immer |
| 16. Ich fühle mich traurig oder niedergeschlagen. | nie | selten | gelegentlich | | oft | immer |
| 17. Ich habe wenig Energie. | nie | selten | gelegentlich | | oft | immer |
| 18. Ich habe Muskelverspannungen im Nacken- und  Schulterbereich. | nie | selten | gelegentlich | | oft | immer |
| 19. Ich habe Kieferschmerzen. | nie | selten | gelegentlich | | oft | immer |
| 20. Mir wird von manchen Gerüchen, wie z.B. Parfüm,  schwindlig und übel. | nie | selten | gelegentlich | | oft | immer |
| 21. Ich muss häufig Wasserlassen. | nie | selten | gelegentlich | | oft | immer |
| 22. Meine Beine fühlen sich unangenehm und ruhelos an,  wenn ich versuche nachts einzuschlafen. | nie | selten | gelegentlich | | oft | immer |
| 23. Ich habe Schwierigkeiten, mich an Dinge zu erinnern. | nie | selten | gelegentlich | | oft | immer |
| 24. Ich erlitt als Kind traumatische Erlebnisse | nie | selten | gelegentlich | | oft | immer |
| 25. Ich habe Schmerzen im Beckenbereich. | nie | selten | gelegentlich | | oft | immer |
| **Gesamtsumme =** | | | |  | | |

| **CENTRAL SENSITIZATION INVENTORY German version (CSI-GE)**  **„Zentraler Sensibilisierungsfragebogen“ TEIL B** |
| --- |

**Hat ein Arzt/eine Ärztin bei Ihnen eine der folgenden Diagnosen gestellt?**

**Bitte kreuzen Sie auf der rechten Seite die passende Antwort zu jeder ärztlichen Diagnose an und geben Sie das Jahr an in dem die Diagnose gestellt wurde.**

|  | NEIN | JA | Jahr der Diagnosestellung |
| --- | --- | --- | --- |
| 1. Restless-Legs-Syndrom (Syndrom der unruhigen Beine) |  |  |  |
| 2. Chronisches Erschöpfungssyndrom (Chronisches  Fatigue Syndrom) |  |  |  |
| 3. Fibromyalgie |  |  |  |
| 4. Kiefergelenks-Funktionsstörung (Craniomandibuläre  Dysfunktion) |  |  |  |
| 5. Migräne oder Spannungskopfschmerz |  |  |  |
| 6. Reizdarmsyndrom (Colon irritabile) |  |  |  |
| 7. Unverträglichkeit gegen verschiedene chemische Substanzen (Multiple Chemical Sensitivity) |  |  |  |
| 8. Nackenverletzung (einschließlich Schleudertrauma) |  |  |  |
| 9. Angst- oder Panikattacken |  |  |  |
| 10. Depression |  |  |  |
