# Supplementary material for: Cross-cultural adaptation and validation of the German Central Sensitization Inventory (CSI-GE)
Source: BMC Musculoskelet Disord. 2021 Aug 18;22:708. doi: 10.1186/s12891-021-04481-5 (PMC8375049; doi:10.1186/s12891-021-04481-5)
Supplement: Supplementary file 4 — Additional file 4: Supplement 4. Visualized pairwise correlations between the CSI-GE part A sum score and each questionnaire. [file 12891_2021_4481_MOESM4_ESM.docx]

**Cross-cultural adaptation and validation of the German Central Sensitization Inventory (CSI-GE)**

**Supplement 4: Visualized pairwise correlations between the CSI-GE part A sum score and each questionnaire**

**M Klute ^a^, M Laekeman ^b^, K Kuss ^c^, F Petzke ^a^, A Dieterich ^d^, A Leha ^e^, R Neblett ^f^, S Ehrhardt ^g^,** J Ulma ^h^, A Schäfer ^i^

^a^ Pain Medicine, Department of Anaesthesiology, University Medical Center Göttingen, Germany

^b^ Physiological Psychology, Otto-Friedrich- University of Bamberg, Germany

^c^ Department of General Practice/Family Medicine, Philipps University Marburg, Germany

^d^ Physiotherapy, Faculty of Health, Safety, Society, Furtwangen University, Germany

^e^ Department of Medical Statistics, University Medical Center Göttingen, Germany

^f^ PRIDE Research Foundation, Dallas, Texas, USA

^g^ Faculty of Social Sciences, City University of Applied Sciences, Bremen, Germany

^h^ Clinic for Pain Medicine Bremen, Rotes-Kreuz-Krankenhaus Bremen, Germany

^i^ Faculty of Social Work and Health, University of Applied Science and Art, Hildesheim, Germany

**Supplement 4: Visualized pairwise correlations between the CSI-GE part A sum score and each questionnaire**

Pairwise correlations between each score and the CSI part A have been calculated. Each panel shows one score in a scatter plot vs CSI part A with a superimposed Loess fit for visual guidance. The subtitles give the pairwise correlation coefficient (Kendal’s τ) and the associated Holm adjusted p value of the test of the null hypothesis of no correlation
